# MicroRNA399 is involved in multiple nutrient starvation responses in rice

**DOI:** 10.3389/fpls.2015.00188

**Published:** 2015-03-24

**Authors:** Bin Hu, Wei Wang, Kun Deng, Hua Li, Zhihua Zhang, Lianhe Zhang, Chengcai Chu

**Affiliations:** ^1^State Key Laboratory of Plant Genomics, National Center for Plant Gene Research (Beijing), Institute of Genetics and Developmental Biology, Chinese Academy of SciencesBeijing, China; ^2^University of Chinese Academy of SciencesBeijing, China; ^3^School of Agriculture, Henan University of Science and TechnologyLuoyang, China

**Keywords:** rice, microRNA399 (miR399), over-expressing plants, gene expression, nutrient starvation response

## Abstract

The increasing evidences have revealed that microRNAs (miRNAs) play significant role in nutrient stress response. Previously, miR399 was documented to be induced by phosphorus (P) starvation and involved in regulating P starvation responses. To further investigate the function of miR399 in rice (*Oryza sativa* L.), we performed GeneChip analysis with OsmiR399 over-expressing plants. Interestingly, our results showed that, besides P starvation responsive genes, the expression of a number of genes involved in iron (Fe), potassium (K), sodium (Na), and calcium (Ca) absorption was dramatically up-regulated in OsmiR399 over-expressing plants. Consistently, the concentrations of Fe, K, Na, and Ca were also increased in OsmiR399 over-expressing plants. The expression of OsmiR399 was also up-regulated by these nutrient starvations, respectively. Moreover, the loss-of-function of *LTN1*, the down-stream target of OsmiR399, also resulted in the increase of multiple metal elements and the up-regulation of the absorption related genes. These results indicated that OsmiR399 participates in the regulation of multiple nutrient starvation responses, which also gives new view on understanding the interaction among different nutrients mediated by miR399.

## Introduction

MicroRNA (miRNA) is a class of 21–24 nucleotides no-coding small RNA, which usually down-regulates the expression of target genes at post-transcription level through mRNA degradation or translational repression. More and more evidences have revealed that miRNAs are involved in regulating multiple biological processes, one of which is the stress adaptive response. In plants, the stress-regulated miRNAs have been identified under different stresses, such as drought, cold, salinity, and nutrient deficiency. A great number of miRNAs, for example, miR396, miR168, miR167, miR165, miR319, miR159, miR394, miR156, miR393, miR171, miR158, and miR169 have been identified to be regulated by drought in Arabidopsis (Liu et al., 2008). In rice, 14 miRNAs (miR159, miR169, miR171, miR319, miR395, miR474, miR845, miR851, miR854, miR896, miR901, miR903, miR1026, and miR1125) are significantly induced while 16 miRNAs are significantly repressed by drought (Zhao et al., 2007; Zhou et al., 2010). The expression of miRNAs is also differentially regulated by salt. In Arabidopsis, miR156, miR158, miR159, miR165, miR167, miR168, miR169, miR171, miR319, miR393, miR394, miR396, and miR397 are up-regulated by salt stress while the expression of miR398 is down-regulated (Liu et al., 2008). The expression of miRNAs under cold or heat stress was also studied in plants. MiR397, miR169, miR165, miR166, miR393, miR396, and miR408 were shown to be up-regulated by cold stress in Arabidopsis (Sunkar and Zhu, 2004; Liu et al., 2008). The expression of miR156, miR159, miR160, miR166, miR168, miR169, miR393, and miR827 is increased, while miR172 is significantly repressed under heat stress in wheat (Xin et al., 2010).

The availability of nutrients, such as sulfur (S), phosphorus (P), copper (Cu), and nitrogen (N), can affect the expression of miRNAs. Cu is an important macronutrient for plants and involved in regulating photosynthesis, oxidative responses, and other physiological processes. Among the miRNAs response to nutrient starvation, miR395 and miR399 are two well documented miRNAs involved in S and P starvation responses, respectively (Takahashi et al., 2000; Maruyama-Nakashita et al., 2003; Fujii et al., 2005; Chiou et al., 2006). MiR398 is induced under Cu limiting condition and its up-regulation subsequently increases the Cu availability (Abdel-Ghany and Pilon, 2008). N starvation induces the expression of miR167a and represses the expression of its target, *ARF8*, which regulates the lateral root initiation (Gifford et al., 2008). In contrast, miR169 is significantly repressed by N starvation while *NFYA*, the target of miR169, is induced (Zhao et al., 2011).

S is a constituent of two amino acids, cysteine and methionine, and therefore, it is an important component of protein. In plants, S is acquired and assimilated mainly in the form of sulfate, which is mediated by sulfate transporters and ATP sulphurylase (APS), respectively. MiR395 is involved in regulating S assimilation by targeting the sulfate transporter gene *SULTR2;1* and APS genes (Takahashi et al., 2000; Maruyama-Nakashita et al., 2003). The expression of miR395 is induced by long-term sulfate deficiency while expression of *SULTR2;1* is repressed. The down-regulation of *SULTR2;1* by sulfate deficiency is only detected in the shoots whereas its mRNA is greatly accumulated in the roots, suggesting that the degradation of *SULTR2;1* by miR395 mainly occurs in the shoots (Takahashi et al., 1997, 2000). In addition to *SULTR2;1*, miR395 is also predicted to target several *APS* genes (*APS1, APS2, APS3*, and *APS4*), two (*APS1* and *APS4*) of which were validated by 5′-RACE (Jones-Rhoades and Bartel, 2004; Allen et al., 2005). APS1, APS3, and APS4 were predicted to be localized at the plastid, while APS2 localized at the cytoplasm (Hatzfeld et al., 2000). Most of the sulfate is reduced in the plastid by APS, which indicates that miR395 is an important regulator of sulfate assimilation in plastids (Rotte and Leustek, 2000).

P is one of the most significant macronutrients for plants, which constitutes many important biological molecules such as nucleotides, phospholipids, and ATP. Inorganic phosphate (Pi) is the major form of available P for plants in the soil. However, in most natural soils, the concentration of Pi is very low and consequently results in the P starvation condition. Accordingly, plants have also evolved P starvation responses to increase Pi availability under this condition (Raghothama, 1999). MiR399 is an important component involved in the P starvation signaling. It consists of 6 (miR399a-f) and 11 (OsmiR399a-k) members in Arabidopsis and rice, respectively. The function of miR399 in P starvation signaling was firstly studied in Arabidopsis. Under P starvation condition, the expression of miR399 is dramatically induced while the expression of its target gene *PHO2* (*UBC24*) is decreased (Fujii et al., 2005; Chiou et al., 2006). Both miR399 over-expressing plants and *pho2* mutant showed Pi over-accumulation in the shoots (Fujii et al., 2005; Chiou et al., 2006). *PHO2* encodes an ubiquitin-conjugating enzyme (E2), which is involved in ubiquitin-mediated protein degradation. Recent work showed that the degradation of PHO1 and PT2, two important Pi transporters controlling Pi homeostasis, is dependent on PHO2, and the ubiquitin ligase (E3) NLA is proved to coordinate with PHO2 to mediate this process (Liu et al., 2012; Lin et al., 2013; Park et al., 2014). In addition, the expression of miR399 is up-regulated by the Myb transcription factor PHR1, which is the key transcription factor activating the expression of P starvation responsive genes (Rubio et al., 2001; Nilsson et al., 2007). In rice, the P starvation signal transduction pathway is conserved with that in Arabidopsis, which mainly consists of OsPHR2, OsmiR399, OsPHO2 (LTN1), and the downstream P starvation responsive genes (Zhou et al., 2008; Hu et al., 2011).

Our previous work revealed that OsmiR399 over-expression resulted in the increase of Pi accumulation in rice (Hu et al., 2011). Here we further showed that the concentration of several other nutrients including iron (Fe), potassium (K), sodium (Na), and calcium (Ca) were also increased in OsmiR399 over-expressing plants and OsmiR399s were coordinately up-regulated by these nutrient starvations, respectively, indicating that miR399 expression responds to multiple nutrient starvations and is involved in the regulation of multi-nutrient absorption.

## Materials and methods

### Plant materials and growth conditions

OsmiR399 over-expressing (ox) plants used in this study including OsmiR399f-ox plants (f1 and f8) and OsmiR399j-ox plants (j6 and j8) were generated in Nipponbare background as previously described (Hu et al., 2011). The *ltn1* mutant was identified from our rice mutant population (Ma et al., 2009) by screening the mutants with leaf senescence phenotype. The *ltn1* mutant was shown to over-accumulate Pi in the leaf, which resulted in the leaf tip necrosis phenotype (Hu et al., 2011).

OsmiR399-ox plants, *ltn1* mutant, and wild type Nipponbare were grown in the 1/2 strength Hoagland solution with minor modification, the full nutrition solution contains 2.5 mM KNO_3_, 1 mM Ca(NO_3_)_2_, 1 mM FeSO_4_-EDTA, 1 mM MgSO_4_, 0.5 mM NH_4_H_2_PO_4_, 20 mM H_3_BO_4_, 5 μM MnCl_2_, 0.38 μM ZnSO_4_, 0.16 μM CuSO_4_, 0.06 μM Na_2_MoO_4_. The nutrient starvation solutions were designed by using 1/2 strength modified Hoagland solution lacking specific elements (P, Fe, Na or Ca). The hydroponic culture was adjusted to pH 6.0 with 2 M NaOH before use and the medium was changed every 3 days. The hydroponic cultured seedlings were grown in an incubator with a 12 h day (30°C)/12 h night (28°C) photoperiod, approximately 200 μmol photons m^−2^s^−1^ light intensity, and approximately 70% humidity. The seedlings were maintained under the treatments for 2-week (after germination to sample harvesting) and the samples under different treatments were collected at the same moment.

### RNA isolation, cDNA preparation, and quantitative RT-PCR

Total RNA was extracted using the TRIzol reagent (Invitrogen). Approximately 2 μg of the total RNA treated with DNase I was used to synthesize the first-strand cDNA using oligo(dT)_18_ as primer. The product of first-strand cDNA was used as the template for the PCR. For qRT-PCR, SYBR Green I was added to the reaction mix and run on a Chromo4 real-time PCR detection system (Bio-Rad, CFX96) according to the manufacturer's instructions. The data were analyzed with Opticon monitor software (Bio-Rad). The expression of miRNAs was investigated by analyzing the expression of their precursors (pri-miRNA). The PCR products of each pri-miRNA were sequenced to confirm the specific amplification of the corresponding pri-miRNA. For the mature miRNA assay in the ox lines, the miRNA First-strand cDNA Synthesis kit (Tiangen, KR201) and the miRNA qPCR Detection kit (Tiangen, KR201) were used according to the manufacturer's instructions. Briefly, the miRNAs are polyadenylated by poly(A) polymerase and subsequently converted into cDNA by reverse transcriptase with oligodT priming. The miRNAs are converted into cDNA by reverse transcriptase using both oligo-dT and random priming. The cDNA is then used for real-time PCR quantification of mature miRNA with the forward miRNA specific primer (with the conserved mature OsmiR399 sequence) and the reverse universal primer. Rice *ACTIN1* was used as the internal control in all analyses. The primers for qRT-PCR are listed in Supplementary Table 1. Each gene expression assay with qRT-PCR was replicated for 3 times.

### Affymetrix ATH1 GeneChip assay

The roots of OsmiR399-ox plants (f8) and Nipponbare cultured in 1/2 Hoagland solution for 2-week were collected for GeneChip analysis. The total RNA was extracted with the TRIzol reagent (Invitrogen) according to the manufacturer's instructions. Affymetrix array service was provided by Capitalbio Corporation, Beijing. The GeneChip data was listed in Supplementary Table 2.

### Metal element concentration determination

Roots and shoots of 2-week-old seedlings grown in 1/2 strength Hoagland solution were dried at 70°C for 48 h in an oven. Exactly 80 mg samples were digested with 13 ml concentrated HNO_3_ and 2 ml 30% H_2_O_2_ at 140°C for 30 min. Digested solution volumes were adjusted to 25 ml with de-ionized water. The metal elements were determined with ICP-OES (PE, Optima 2000 DV).

## Results

### OsmiR399 participates in regulating multiple nutrient absorption genes

To investigate how OsmiR399 regulates the expression of downstream genes, we performed GeneChip analysis using OsmiR399-ox plants (f8) and its corresponding wild type Nipponbare. The materials of roots were collected and assayed with Affymetrix GeneChip. As expected, the expression of Pi absorption-related genes was significantly increased in the roots of OsmiR399-ox plants (Table 1). Interestingly, several genes probably involved in metal element absorption were also largely up-regulated in OsmiR399-ox plants (Table 1), including the genes encoding nicotianamine synthase (OsNAS1 and OsNAS2), potassium transporter (KT), sodium transporter (NaT), and calcium channel (CaC), which gives the hint for the connection between miR399 and multiple nutrient absorption in rice.

**Table 1:** **The up-regulated genes involved in nutrient absorption in OsmiR399-ox plants (f8) from GeneChip assay**.

**Gene name**	**Loc. No**.	**Fold change**	***P*-value**
**P ABSORPTION RELATED GENES**			
Putative high-affinity phosphate transporter	LOC_Os08g45000	3.9	0.00002
Putative inorganic phosphate transporter	LOC_Os10g30770	2.6	0.00002
Phosphate transporter	LOC_Os03g05640	2.3	0.00002
Putative phosphate transporter	LOC_Os10g30790	1.5	0.00004
Ribonuclease	LOC_Os08g33710	1.5	0.00002
Putative purple acid phosphatase	LOC_Os01g56880	2.4	0.00002
Partial mRNA for phospholipase C	LOC_Os12g37560	1.3	0.001651
Secretory acid phosphatase precursor	LOC_Os12g44020	1.2	0.00003
Secretory acid phosphatase precursor	LOC_Os12g44020	1.2	0.00003
**METAL ELEMENTS ABSORPTION RELATED GENES**			
Nicotianamine synthase (OsNAS1)	LOC_Os03g19420	2.1	0.000033
Nicotianamine synthase (OsNAS2)	LOC_Os03g19427	2.8	0.000041
Potassium transporter (KT)	LOC_Os02g49760	1.5	0.00002
Sodium transporter (NaT)	LOC_Os05g31730	2.8	0.000035
Calcium-transporting ATPase (CaC)	LOC_Os01g71240	3.1	0.00002

*The P-value was determined by One-Way ANOVA. The fold change represents the increased folds of gene expression in OsmiR399-ox plants compared with wild type*.

**Figure 1:**
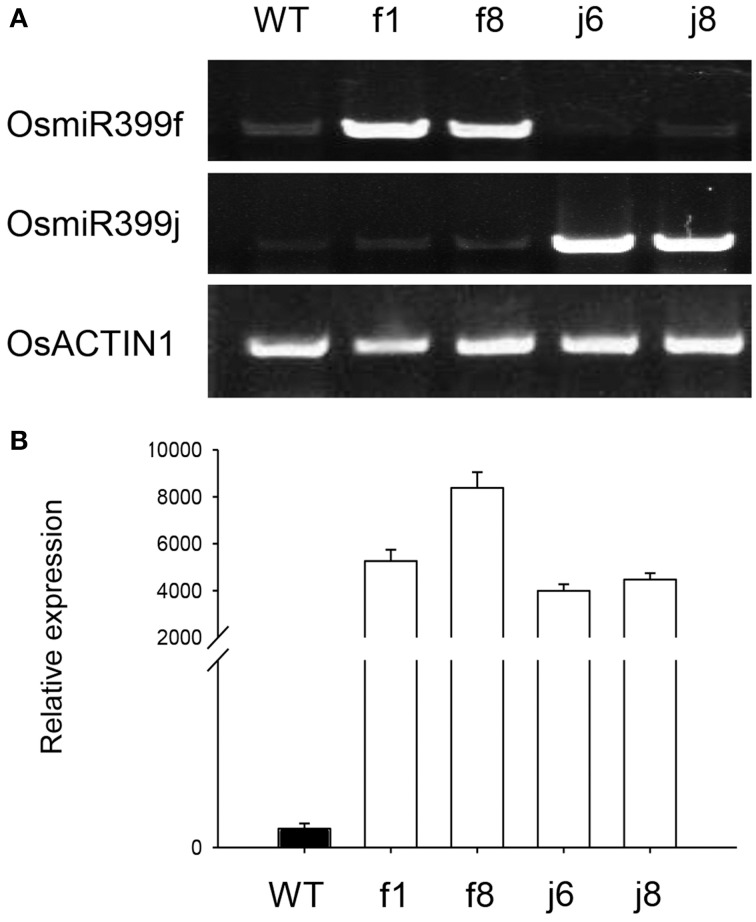
**OsmiR399 was over-expressed in the OsmiR399-ox plants. (A)** The expression assay of the pri-miRNA (OsmiR399f and OsmiR399j) in the shoots of OsmiR399-ox plants (f1, f8, j6, and j8) and wild type (WT) with semi-quantitative RT-PCR. **(B)** The mature OsmiR399 abundance assay in the shoots of OsmiR399-ox plants (f1, f8, j6, and j8) and wild type (WT) with qRT-PCR. The mature miRNA abundance in WT was set as 1.

**Figure 2:**
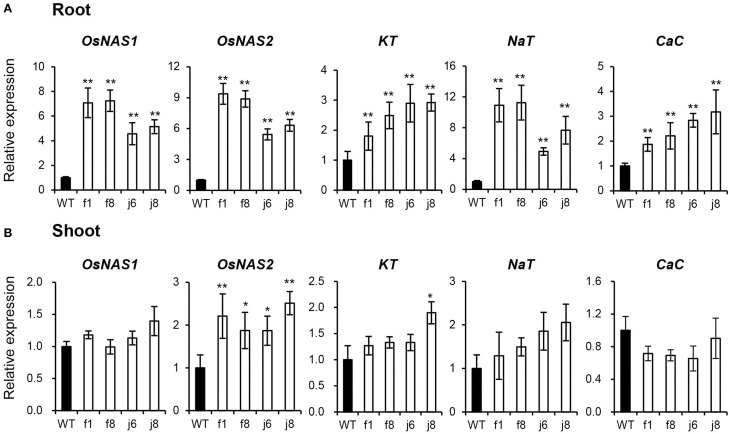
**Metal element absorption-related genes were up-regulated in OsmiR399-ox plants**. The transcript expression of *OsNAS1, OsNAS2, KT, NaT*, and *CaC* in roots **(A)** and shoots **(B)** of WT (wild type, Nipponbare) and OsmiR399-ox plants (f1, f8, j6, and j8). The transcript expression was determined by quantitative RT-PCR (qRT-PCR). Values are the means ± SD (*n* = 3). The asterisks indicate the significant difference determined by student *t*-test: ^*^P < 0.05, ^**^P < 0.01. The transcript expression of these genes in WT was set as 1.

To confirm the result obtained from GeneChip, four independent OsmiR399-ox lines including f1, f8, j6, and j8 were further analyzed. Both the pri-miRNA and mature miRNA were significantly over-expressed in theses ox lines (Figure 1). The expression assay of the metal absorption related genes in these ox lines showed that *OsNAS1, OsNAS2, KT, NaT*, and *CaC* were significantly increased in the roots of OsmiR399-ox lines (Figure 2A), whereas in shoots, except *OsNAS2*, no significant alteration was observed in the expression of these genes in OsmiR399-ox lines (Figure 2B). These data indicates that regulation of these genes by OsmiR399 occurs mainly in the roots.

### Fe, K, Na, and Ca concentrations were increased in OsmiR399-ox plants

**Figure 3:**
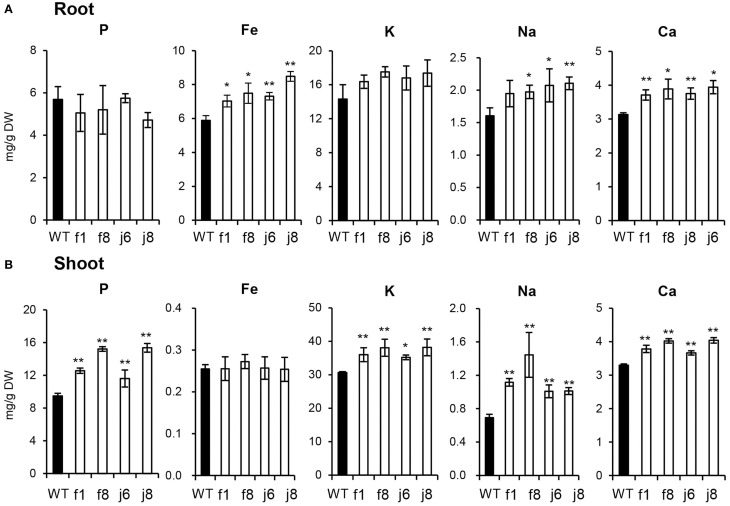
**P, Fe, K, Na, and Ca concentrations were increased in OsmiR399-ox plants**. P, Fe, K, Na, and Ca concentrations were determined in the roots**(A)** and shoots **(B)** of WT (wild type, Nipponbare) and OsmiR399-ox plants (f1, f8, j6, and j8). Values are the means ± SD (*n* = 5). The asterisks indicate the significant difference determined by student *t*-test: ^*^P < 0.05, ^**^P < 0.01.

The up-regulation of the related genes suggested that the absorption of multiple nutrients is increased by OsmiR399 over-expression. Thus, the concentrations of multiple elements were further determined in OsmiR399-ox plants (f1, f8, j6, and j8) and the wild-type Nipponbare. The P concentration was largely increased in the shoots of all OsmiR399-ox plants while no significant alteration was found in the roots (Figure 3). In contrast, the Fe concentration was only increased in the roots of OsmiR399-ox plants (Figure 3A). The K concentration was also specifically increased in the shoots, and Na and Ca concentrations were increased in both roots and shoots of OsmiR399-ox plants (Figure 3). These results provided further evidence that OsmiR399 is involved in regulating the absorption of multiple nutrients, and also suggested that the mechanisms of regulation by OsmiR399 differ among different nutrients.

### OsmiR399 can be induced by Fe, K, Na, or Ca starvation

**Figure 4:**
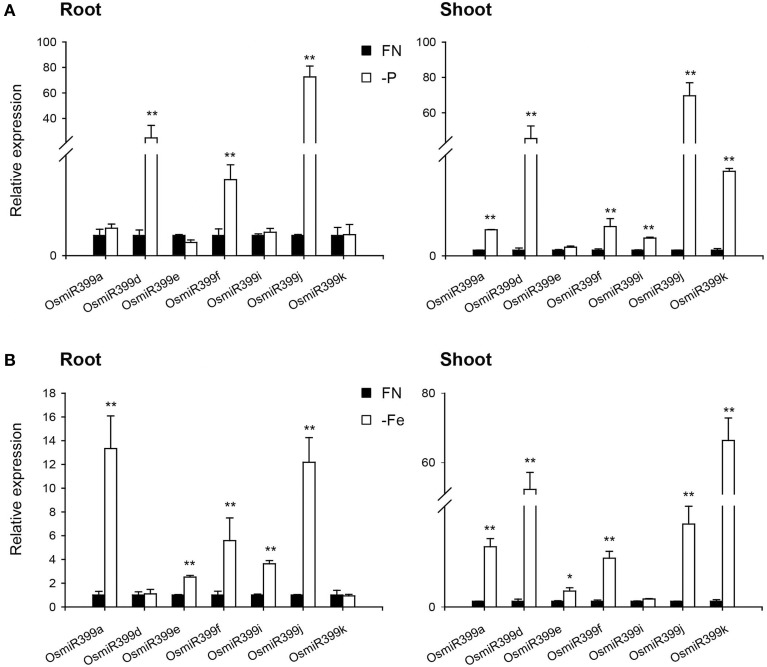
**OsmiR399s were induced by P or Fe starvation. (A)** The transcript expression assay of OsmiR399 under P starvation (-P). **(B)** The transcript expression assay of OsmiR399 under Fe starvation (-Fe). The expression of 7 OsmiR399 members (OsmiR399a, OsmiR399d, OsmiR399e, OsmiR399f, OsmiR399i, OsmiR399j, OsmiR399k) were examined by analyzing the expression of their precursors (pri-miRNA). FN, full nutrition condition. The transcript expression was determined by qRT-PCR. Values are the means ± SD (*n* = 3). The asterisks indicate the significant difference determined by student *t*-test: ^*^P < 0.05, ^**^P < 0.01. The transcript expression of OsmiR399 under FN was set as 1.

**Figure 5:**
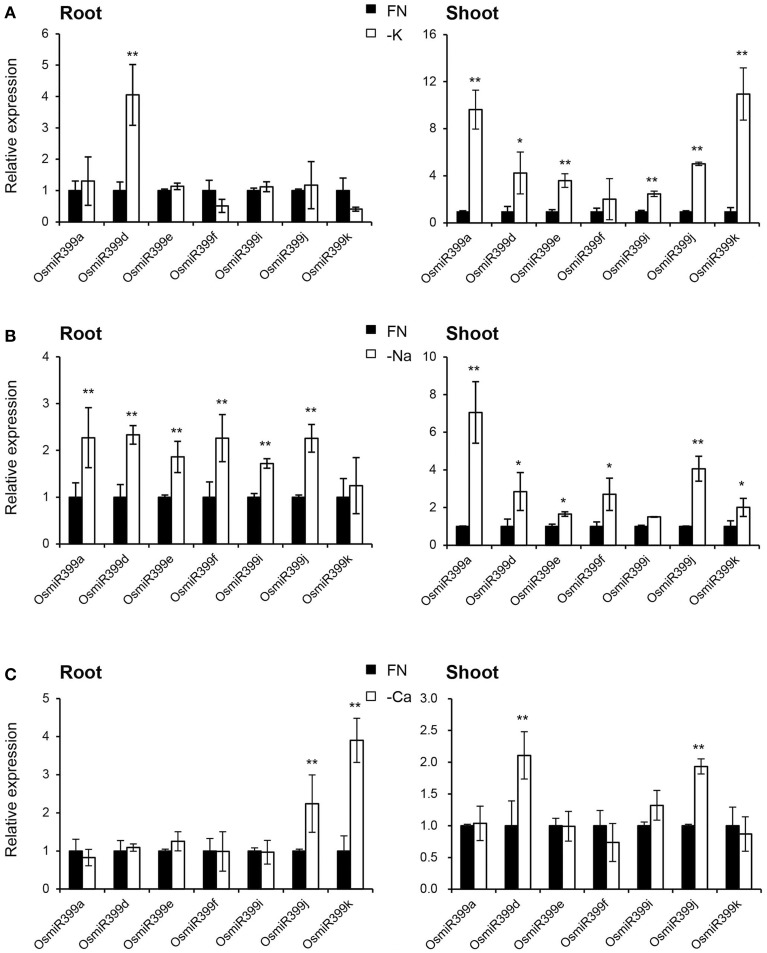
**OsmiR399s were induced by K, Na or Ca starvation. (A)** The transcript expression assay of OsmiR399 under K starvation (-K). **(B)** The transcript expression assay of OsmiR399 under Na starvation (-Na). **(C)** The transcript expression assay of OsmiR399 under Ca starvation (-Ca). The expression of seven OsmiR399 members (OsmiR399a, OsmiR399d, OsmiR399e, OsmiR399f, OsmiR399i, OsmiR399j, OsmiR399k) were examined by analyzing the expression of their precursors (pri-miRNA). FN, full nutrition condition. The transcript expression was determined by qRT-PCR. Values are the means ± SD (*n* = 3). The asterisks indicate the significant difference determined by student *t*-test: ^*^P < 0.05, ^**^P < 0.01. The transcript expression of OsmiR399 under FN was set as 1.

As over-expression of OsmiR399 resulted in increased absorption of multiple nutrients, we further investigated whether OsmiR399 is responsive to these nutrient starvations. Pri-miRNA is the primary transcript of the mature miRNA. The mature miRNAs have very high sequence similarity between different members while the sequence similarity of the pri-miRNAs is relatively low except in the mature miRNA region. This sequence heterology can help to detect the expression of different members of miR399 family via analyzing the expression of the corresponding pri-miRNAs. Thereby, the expression of seven OsmiR399 members (OsmiR399a, OsmiR399d, OsmiR399e, OsmiR399f, OsmiR399i, OsmiR399j, and OsmiR399k) was examined by analyzing the expression of their corresponding pri-miRNAs with qRT-PCR. Consistent with the previous result, most members of OsmiR399 can be induced by P starvation in the shoots, except three members (OsmiR399d, OsmiR399f, and OsmiR399j) which were significantly induced in the roots (Figure 4A). To our surprise, most OsmiR399s also can be significantly induced by Fe starvation in both roots and shoots and the induced level is much higher in shoots than in roots (Figure 4B). Notably, the induction of OsmiR399 by Fe starvation is almost comparable to that by P starvation in the shoots, indicating OsmiR399 is an important component involved in Fe starvation responses. In shoots, most members of OsmiR399 were induced by K starvation, especially OsmiR399a and OsmiR399k, whereas in roots, only OsmiR399d was up-regulated by K starvation (Figure 5A). Under Na starvation, the up-regulation of OsmiR399 was also observed in both shoots and roots. However, only two specific OsmiR399 transcripts were induced by Ca starvation in roots (OsmiR399j and OsmiR399k) and shoots (OsmiR399d and OsmiR399j), respectively (Figures 5B,C). It is noted that the induction of OsmiR399 under K, Na, or Ca starvation was much less than that under P starvation. The above results demonstrated that OsmiR399 is an important component involved in multiple nutrient starvation responses.

### LTN1 is involved in regulation of multiple nutrients absorption

**Figure 6:**
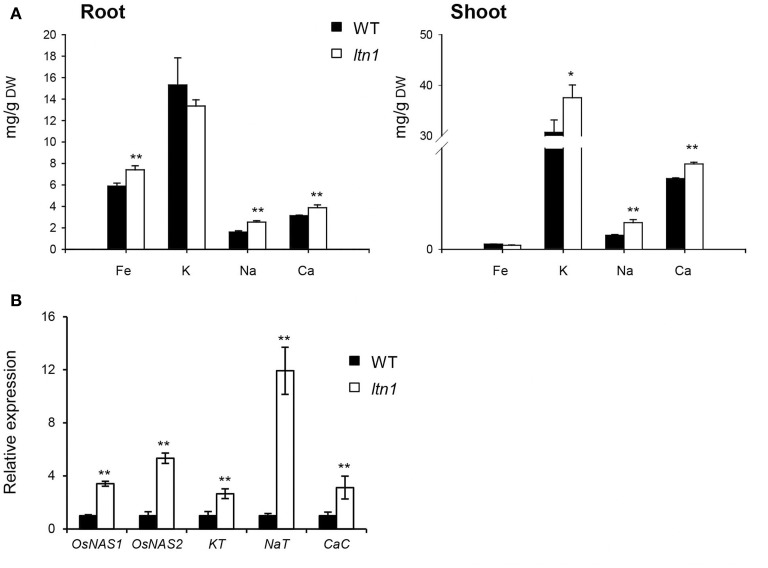
**Fe, K, Na, and Ca concentrations and the absorption related genes expression analyses in WT (wild type, Nipponbare) and *ltn1* mutant. (A)** Fe, K, Na, and Ca concentrations in the roots and shoots of WT and *ltn1* mutant. Values are the means ± SD (*n* = 5). **(B)** The transcript expression assay of *OsNAS1, OsNAS2, KT, NaT*, and *CaC* in the roots of WT and *ltn1* mutant. The transcript expression was determined by qRT-PCR. Values are the means ± SD (*n* = 3). The asterisks indicate the significant difference determined by student *t*-test: ^*^P < 0.05, ^**^P < 0.01.

As *LTN1* is the downstream target of OsmiR399 (Hu et al., 2011), it is possible that LTN1 is also involved in the regulation of multiple nutrient absorption. To verify this speculation, the nutrient concentrations were determined and the expression of the related genes was examined in the *ltn1* mutant. The results showed that the Fe concentration was increased in the roots of *ltn1* mutant (Figure 6A), which is consistent with the result from OsmiR399-ox plants. The concentration of K was only increased in the shoots, while Na and Ca concentrations were increased in both roots and shoots of *ltn1* mutant (Figure 6A). Consistently, the transcript expression of *OsNAS1, OsNAS2, KT, NaT*, and *CaC* was also significantly increased in the roots of *ltn1* mutant (Figure 6B). These results indicated that LTN1 is involved in the regulation of multiple nutrient absorptions.

## Discussion

Previous work showed that miR399 is a significant component mediating P starvation responses. In this work, our data further demonstrated that miR399 is also involved in regulating multiple nutrient starvation responses. The OsmiR399-ox plants displayed increased expression of genes involved in metal element absorption. *OsNAS1* and *OsNAS2*, the nicotianamine synthase genes, were up-regulated in OsmiR399-ox plants. Previous work suggested that OsNAS1 and OsNAS2 mediate the long-distance transport of Fe (Inoue et al., 2003), while our results indicated that their up-regulation may also lead to increased Fe accumulation in roots. The expression of the K and Na transporter genes, *KT* and *NaT*, was also elevated in OsmiR399-ox plants, resulting in the increase of K and Na concentrations. The increase of Ca concentration in OsmiR399-ox plants was possibly associated with the enhanced expression of the calcium channel encoding gene *CaC*. In Arabidopsis, it was reported that miR399 is specifically induced by P starvation, but not by N, S, or K starvation (Fujii et al., 2005). Our results revealed that, besides P starvation, the expression of OsmiR399 is also responsive to Fe, K, Na, or Ca starvation. However, except Fe starvation, the induction of OsmiR399 by other metal element starvations was significantly lower than that by P starvation. Since there is a strong interaction between different nutrients, it is possible that the deficiencies of these metal elements result in the repression of Pi uptake. Whether OsmiR399 is directly induced by these metal element starvations or by P starvation caused by these nutrient deficiencies needs to be further investigated.

**Figure 7:**
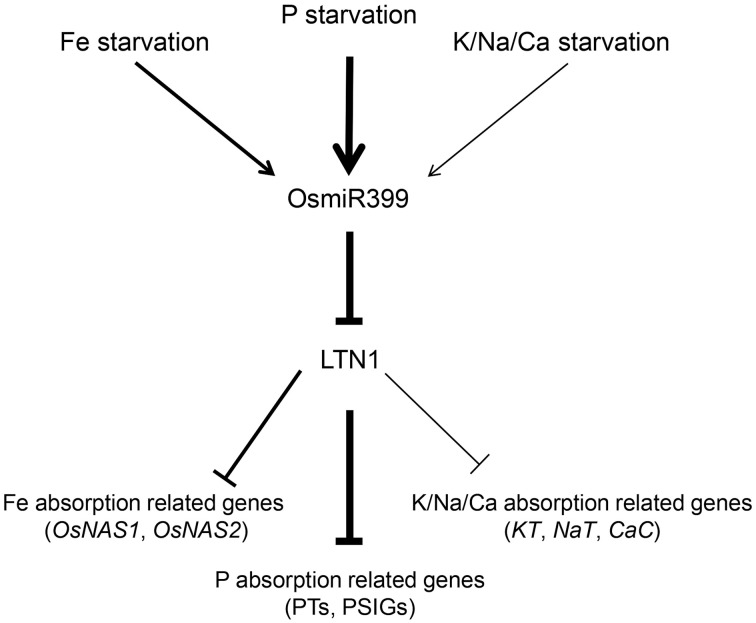
**Working model of multiple nutrient starvation responses mediated by OsmiR399 and LTN1 in rice**. P, Fe, K, Na, or Ca starvations induce the expression of OsmiR399. The up-regulation of OsmiR399 results in the repression of *LTN1*, which subsequently activates the expression of multiple nutrient absorption-related genes including Pi transporter genes (PTs), P starvation-induced genes (PSIGs), Fe absorption-related genes (*OsNAS1, OsNAS2*), and K/Na/Ca absorption-related genes (*KT, NaT*, and *CaC*). The thick and thin arrows indicate the strong and weak regulation, respectively.

Since miRNAs usually function as the negative regulators (Fujii et al., 2005; Chiou et al., 2006; Hu et al., 2011), it is reasonable to speculate that these up-regulated genes in OsmiR399-ox plants are probably under the control of LTN1. Our previous work also showed that, in the roots of *ltn1* mutant, Fe concentration was increased while the genes involved in Fe assimilation including *OsNAS1* and *OsNAS2* were up-regulated (Hu et al., 2011), further suggesting that OsmiR399 regulates metal element absorption through repressing the expression of *LTN1*. In this work, we showed that LTN1 also regulates K, Na, and Ca absorption. These results indicated that OsmiR399 and LTN1 possibly play a pivotal role in regulating multiple nutrient responses in plants. Based on these results, we proposed the following model (Figure 7): P, Fe, K, Na, or Ca starvation induces the expression of OsmiR399, the up-regulation of OsmiR399 results in the repression of *LTN1*, which subsequently activates the expression of multiple nutrient absorption-related genes including Pi transporter genes (PTs), P starvation-induced genes (PSIGs), Fe absorption-related genes (*OsNAS1, OsNAS2*), and K/Na/Ca absorption-related genes (*KT, NaT*, and *CaC*). The up-regulation of these genes will enhance the absorption of multiple nutrients, which finally improves the adaptive ability of plants under nutrient starvation condition. Although P starvation can directly induce the expression of miR399, it is unknown whether induction of miR399 by other nutrient starvation is direct or indirect. As OsmiR399 is the target of OsPHR2 (Zhou et al., 2008), it is possible that OsPHR2 is involved in this process. Investigation of the expression of these nutrient absorption related genes and the concentrations of multiple nutrients in *OsPHR2*ox/RNAi transgenic plants will help to complete the signaling pathway of nutrient starvation responses mediated by miR399.

## Conclusion

The miR399 was previously demonstrated to mediate the P starvation signal transduction in both Arabidopsis and rice. Our work further showed that miR399 is also involved in regulating absorption of multiple nutrients and its expression is regulated by the status of these nutrients in rice. This result extends our knowledge on the function of miR399 in plant nutrition and also gives an evidence that miR399 probably is an important regulator for multiple nutrient interactions.

### Conflict of interest statement

The authors declare that the research was conducted in the absence of any commercial or financial relationships that could be construed as a potential conflict of interest.
