# Supplementary material for: MicroRNA399 is involved in multiple nutrient starvation responses in rice
Source: Front Plant Sci. 2015 Mar 24;6:188. doi: 10.3389/fpls.2015.00188 (PMC4371656; doi:10.3389/fpls.2015.00188)
Supplement: Supplementary file 1 [file Table1.DOC]

|  | Forward (5’-3’) | Reverse (5’-3’) |
| --- | --- | --- |
| OsNAS1( LOC_Os03g19420) | ttcgtacgtgttgtttggga | gggtcctcattgattgctgt |
| OsNAS2 ( LOC_Os03g19427) | gaggctatccgtctgagtgc | aaccccacagaagcacaaac |
| KT ( LOC_Os02g49760) | ttgtctgcgtctggcttctct | aaccacctgtttgggtcttctt |
| NaT ( LOC_Os05g31730) | agctcatcgtgctcaacatc | cattgagctccacgtcatc |
| CaC ( LOC_Os01g71240) | agagcctcctccagatcaat | atcttctccatctccctgct |
| OsmiR399a | gctggaaatgatgctggtagc | ctcctttggcacgagatctgt |
| OsmiR399d | ggtggcctttgatagaccatca | gcaggccgttttggtgaat |
| OsmiR399e | gtgagtcttccttggcagtg | gcagcggtgaactggtga |
| OsmiR399f | ggcagaggtgatcagattgca | ggcaaatctcctttggcagag |
| OsmiR399i | gcagttctcctctggcatg | ttggcagggaaaggctag |
| OsmiR399j | ggagcatgtgaagtcttttgtagc | ggcaactctcctttggcaga |
| OsmiR399k | tgttgcagttcatcatcg | ttcctttggcagtagtct |
| OsmiR399-mature | tgccaaaggagatttgccc |  |
| OsACTIN1 ( LOC_Os03g50885) | accattggtgctgagcgttt | cgcagcttccattcctatgaa |

**Supplemental Table 1.** The primers used for qRT-PCR and semi-quantitative RT-PCR.
